# We Have an Inflation of Review Papers—for what Are Reviews Good?

**DOI:** 10.3389/fpls.2016.00088

**Published:** 2016-02-08

**Authors:** Ingo Schubert

**Affiliations:** Breeding Research, Karyotype Evolution, Leibniz Institute of Plant Genetics and Crop Plant Researcdh (IPK)Gatersleben, Germany

**Keywords:** science writing, citation behavior, impact factor, benefit of review papers, quality of review papers

In recent years more and more reviews have been published, and journal editors aggressively woo authors to submit such scripts. What are the reasons? One is that the flood of original scientific papers is overwhelming. Many researchers feel overburdened to read all original papers they wish to read, in particular, papers on the edge of the own research field. This generates a demand for good reviews from the side of researchers. The motivation for publishers and authors of reviews, however, is not solely to serve the needs of researchers. Often reviews get cited more frequently than original research articles. This is good for the impact factor of the publishing journal, and doubly beneficial for the review authors.

However, reviews also have underbellies. The most obvious one is if they are cited without the remark “for review,” then readers unfamiliar with details of the topic may believe the review author did the corresponding original research. This comes close to scientific misconduct. Even if cited as “for review,” the honor (and citation!) is lost for the authors of the original work which actually deserved the citation. An embarrassing claim by some journals is to restrict citations within review papers to very recent time periods, neglecting that not all essential contributions to scientific progress were done during the last 5 years.

Last, but not least, the increasing number of reviews generates a similar problem as original papers do, namely: what to read and what to cite? This effect is then amplified as many reviews on the same topic may appear nearly at the same time, due to the competition of journals for review papers. As a consequence, many reviews are neither of high quality nor sufficiently novel.

What are the hallmarks of a good review? In my opinion, there are two classes of useful reviews: (i) comprehensive and well-ordered literature surveys and (ii) (even better) surveys providing, in addition, novel aspects and views resulting from the synopsis of the original papers within a field. Reviews that do not meet such criteria are arguably not worth publishing (but can hardly be rejected if invited).

Thus, I encourage potential review authors not to take the bait simply to have one more (hopefully well-cited) paper, if they do not feel certain they can meet the criteria for a good review, or if they are aware of a similar paper recently published or in press. This might be a way to avoid contamination of scientific literature with less valuable review papers and reduce the uncertainty of researchers regarding which of the many similar reviews to read and to cite in the own papers. In summary, my recommendation is to write a review only if you feel your review could add significance to the original papers published in the field of interest.

## Author contributions

The author confirms being the sole contributor of this work and approved it for publication.

### Conflict of interest statement

The author declares that the research was conducted in the absence of any commercial or financial relationships that could be construed as a potential conflict of interest.

